# Neuroinflammation and Functional Connectivity in Alzheimer's Disease: Interactive Influences on Cognitive Performance

**DOI:** 10.1523/JNEUROSCI.2574-18.2019

**Published:** 2019-09-04

**Authors:** L. Passamonti, K.A. Tsvetanov, P.S. Jones, W.R. Bevan-Jones, R. Arnold, R.J. Borchert, E. Mak, L. Su, J.T. O'Brien, J.B. Rowe

**Affiliations:** ^1^Istituto di Bioimmagini e Fisiologia Molecolare (IBFM), Consiglio Nazionale delle Ricerche (CNR), 20090, Milano, Italy,; ^2^Departments of Clinical Neurosciences,; ^3^Psychiatry, University of Cambridge, Cambridge CB2 0SZ, United Kingdom, and; ^4^Cognition and Brain Sciences Unit, Medical Research Council, Cambridge CB2 7EF, United Kingdom

**Keywords:** [^11^C]PK11195, Alzheimer's disease, functional connectivity, independent component analysis, neuroinflammation, PET

## Abstract

Neuroinflammation is a key part of the etio-pathogenesis of Alzheimer's disease (AD). We tested the relationship between neuroinflammation and the disruption of functional connectivity in large-scale networks, and their joint influence on cognitive impairment. We combined [^11^C]PK11195 positron emission tomography (PET) and resting-state functional magnetic resonance imaging (rs-fMRI) in 28 patients (12 females/16 males) with clinical diagnosis of probable AD or mild cognitive impairment with positive PET biomarker for amyloid, and 14 age-, sex-, and education-matched healthy controls (8 females/6 males). Source-based “inflammetry” was used to extract principal components of [^11^C]PK11195 PET signal variance across all participants. rs-fMRI data were preprocessed via independent component analyses to classify neuronal and non-neuronal signals. Multiple linear regression models identified sources of signal covariance between neuroinflammation and brain connectivity profiles, in relation to the diagnostic group (patients, controls) and cognitive status.

Patients showed significantly higher [^11^C]PK11195 binding relative to controls, in a distributed spatial pattern including the hippocampus, frontal, and inferior temporal cortex. Patients with enhanced loading on this [^11^C]PK11195 binding distribution displayed diffuse abnormal functional connectivity. The expression of a stronger association between such abnormal connectivity and higher levels of neuroinflammation correlated with worse cognitive deficits.

Our study suggests that neuroinflammation relates to the pathophysiological changes in network function that underlie cognitive deficits in Alzheimer's disease. Neuroinflammation, and its association with functionally-relevant reorganization of brain networks, is proposed as a target for emerging immunotherapeutic strategies aimed at preventing or slowing the emergence of dementia.

**SIGNIFICANCE STATEMENT** Neuroinflammation is an important aspect of Alzheimer's disease (AD), but it was not known whether the influence of neuroinflammation on brain network function in humans was important for cognitive deficit. Our study provides clear evidence that *in vivo* neuroinflammation in AD impairs large-scale network connectivity; and that the link between neuro inflammation and functional network connectivity is relevant to cognitive impairment. We suggest that future studies should address how neuroinflammation relates to network function as AD progresses, and whether the neuroinflammation in AD is reversible, as the basis of immunotherapeutic strategies to slow the progression of AD.

## Introduction

Neuroinflammation plays a key role in the etio-pathogenesis of Alzheimer's disease (AD) and other neurodegenerative disorders (Edison et al., 2008; Fernández-Botran et al., 2011; Fan et al., 2015; Stefanetti et al., 2016). Preclinical models (Heppner et al., 2015; Hoeijmakers et al., 2016; Villegas-Llerena et al., 2016; Li et al., 2018; Wang et al., 2018), and research in humans (Fernández-Botran et al., 2011; Edison et al., 2013; Fan et al., 2015; Stefaniak and O'Brien, 2016; Passamonti et al., 2018), demonstrate that microglia, the brain's innate immune system, are activated in AD and other neurodegenerative diseases. Furthermore, genetic association studies have demonstrated a link between AD and polymorphisms or mutations in genes linked to immune response (Villegas-Llerena et al., 2016). Although the mechanisms and mediators of inflammatory risk in AD are not fully understood, synaptic and neuronal injury may arise from the release of cytokines and proinflammatory molecules such as interleukin-1β and TGF-β (Fernández-Botran et al., 2011), or direct microglial injury to synapses (Hong and Stevens, 2016; Hong et al., 2016). These, in turn, impair synaptic function, network communication, and may accelerate neurodegeneration and synaptic loss (Heppner et al., 2015; Hoeijmakers et al., 2016; Villegas-Llerena et al., 2016; Li et al., 2018; Wang et al., 2018).

Clinical studies of neuroinflammation in dementia have exploited positron emission tomography (PET) ligands that bind to the mitochondrial translocator protein (TSPO) in activated microglia (Cagnin et al., 2001; Gerhard et al., 2006a,b; Edison et al., 2008, 2013; Fan et al., 2015; Passamonti et al., 2018). For example, relative to controls, patients with AD have higher [^11^C]PK11195 binding in the hippocampus, other medial-temporal lobe regions, and posterior cortices such as the precuneus (Passamonti et al., 2018).

These findings raise the possibility of immunotherapeutic strategies to prevent or slow the progression of AD. However, key issues remain to be resolved before such therapeutic strategies can be realized. For example, it is necessary to show how neuroinflammation is linked to cognitive deficits. A critical and unanswered question is whether regional neuroinflammation changes the functional connectivity of large-scale networks. Such large-scale neural networks represent an intermediate phenotypic expression of pathology in many diseases that can be non-invasively quantified with resting-state functional magnetic resonance imaging (fMRI). A challenge is that neither the anatomical substrates of cognition nor the targets of neurodegenerative disease are mediated by single brain regions: they are in contrast distributed across multivariate and interactive networks.

We thus undertook a multimodal and multivariate neuroimaging study to combine [^11^C]PK11195 quantification of distributed neuroinflammation with resting-state functional imaging in patients at different stages of AD. We used “source-based inflammetry” (SBI; analogous to “volumetry”) to reduce the dimensionality (i.e., complexity) of the neuroinflammation signal, and used multiple linear-regression models to associate neuroinflammation, functional network connectivity, and cognition.

We tested two hypotheses: (1) that spatially distributed neuroinflammation related to significant changes in large-scale functional connectivity in patients with AD, relative to controls. (2) That the relationship between neuroinflammation and abnormal functional connectivity mediates cognitive deficit in AD.

## Materials and Methods

### 

#### Participants

The study was conducted in the context of the Neuroimaging of Inflammation in MemoRy and Other Disorders (NIMROD) study (Bevan-Jones et al., 2017). We included 14 patients meeting clinical diagnostic criteria for probable AD (McKhann et al., 2011), and 14 patients with mild cognitive impairment (MCI); (12 females and 16 males in total) defined by: (1) a mini-mental score examination MMSE >24/30; (2) memory impairment at least 1.5 standard deviation (SD) below that expected for age and education (Petersen et al., 1999); and (3) biomarker evidence of amyloid pathology [positive Pittsburgh Compound-B PET scan (MCI+); Okello et al., 2009]. We combined patients with clinical AD and MCI+ on the basis that these two groups represent a continuum of the same clinical spectrum (Okello et al., 2009).

Fourteen age-, sex-, and education-matched healthy controls (8 females, 6 males) with no history of major psychiatric or neurological illnesses, head injury, or any other significant medical comorbidity were also recruited. All participants were aged >50 years, with premorbid proficiency in English for cognitive testing, and had no acute infectious or chronic symptomatic systemic inflammatory disorder (e.g., lupus, rheumatoid arthritis, etc.), or contraindications to MRI. Patients were identified from the Cambridge University Hospitals NHS Trust Memory Clinics and the Dementias and Neurodegenerative Diseases Research Network (DeNDRoN), whereas healthy controls were recruited via DeNDRoN. All participants had mental capacity and gave written consent in accordance with the Declaration of Helsinki. The study was approved by the local research ethics committee.

#### Clinical and cognitive assessment

Clinical indices of cognitive deficit included Mini Mental State Examination (MMSE), Addenbrooke's Cognitive Examination-Revised (ACE-R), and Rey auditory verbal learning test (RAVLT). The demographic and neuropsychological measures are reported in Table 1. A principal component analysis (PCA) on the total MMSE, ACE-R, and RAVLT scores was conducted to reduce the dimensionality of the cognitive deficit into one latent variable, which summarized the largest portion of shared variance as the first principal component.

**Table 1.** Participant details (mean, with SD and range in parentheses) and group differences by χ^2^ test, one-way ANOVA, or independent samples *t* test

Demographic and clinical data	AD/MCI+ (*N* = 28)	Controls (*N* = 14)	AD/MCI+ < Controls
Sex, females/males	12/16	8/6	NS
Age, years (SD, range)	72.7 (±8.5, 53–86)	68.3 (±5.4, 59–81)	NS
Education, years (SD, range)	12.9 (±3.0, 10–19)	14.1 (±2.7, 10–19)	NS
MMSE (SD, range)	25.6 (±2.2, 21–30)	28.8 (±1.0, 27–30)	t = 4.9, *p* < 0.0001
ACE-R (SD, range)	78.9 (±7.7, 62–91)	91.6 (±5.3, 79–99)	t = 5.5, *p* < 0.0001
RAVLT (SD, range)	1.5 (±1.6, 0–6)	9.6 (±3.2, 3–15)	t = 10.8, *p* < 0.0001

NS, Not significant with *p* > 0.05 (uncorrected).

#### Experimental design

##### Structural and fMRI protocols and preprocessing.

Structural and functional MRI were performed using a 3-tesla Siemens Tim Trio scanner with a 32-channel phased-array head coil. A T1-weighted magnetization-prepared rapid gradient-echo image was acquired with repetition time = 2300 ms, echo time = 2.98 ms, matrix = 256 × 240, in-plane resolution of 1 × 1 mm, 176 slices of 1 mm thickness, inversion time = 900 ms and flip angle = 9 degrees. The coregistered T1 images were used in a single-channel segmentation to extract probabilistic maps of six tissue classes: gray matter, white matter, cerebro-spinal fluid (CSF), bone, soft tissue, and background noise. The native-space gray-matter and white-matter images were submitted to diffeomorphic registration to create group template images (Ashburner, 2007). The template was normalized to the Montreal Neurological Institute (MNI) template using a 12-parameter affine transformation. After applying the normalization parameters from the T1 stream to warp preprocessed functional images into MNI space, the normalized images were smoothed using an 8 mm Gaussian kernel. An estimate of total gray matter, used in between-subject analysis as a covariate of no interest, was calculated as the median gray-matter tissue intensity in a group mask based on voxels with gray-matter tissue probability of 0.3 across all individuals. Resting-state multi-echo functional imaging was performed for 11 min. A total of 269 echoplanar image volumes were acquired with repetition time = 2430 ms, echo times = 13.00, 30.55, and 48.10 ms, matrix = 64 × 64, in-plane resolution of 3.75 × 3.75 mm, 34 slices of 3.8 mm thickness with an inter-slice gap of 0.38 mm, and a generalized autocalibrating partial parallel acquisition (GRAPPA) imaging with an acceleration factor of 2 and bandwidth = 2368 Hz/pixel. The first six volumes were discarded to eliminate saturation effects and achieve steady-state magnetization. Pre-processing of resting-state data used the Multi-Echo Independent Components Analysis (ME-ICA) pipeline, which uses independent component analysis to classify blood oxygenation level-dependent (BOLD) and non-BOLD signals based on the identification of linearly dependent and independent echo-time related components (https://wiki.cam.ac.uk/bmuwiki/MEICA; Kundu et al., 2013). This provides an optimal approach to correct for movement-related and non-neuronal signals, and is therefore particularly suited to our study, in which systematic differences in head position might have been expected between groups. After ME-ICA, the data were smoothed with 5.9 mm full-width half-maximum Gaussian kernel.

The location of the key brain regions in each network was identified by spatial independent component analysis (ICA) using the Group ICA of fMRI Toolbox (Calhoun et al., 2001) in an independent dataset of 298 age-matched healthy individuals from the population-based cohort in the Cambridge Centre for Aging and Neuroscience (Cam-CAN; Shafto et al., 2014). Details about pre-processing and node definition are published previously (Tsvetanov et al., 2016). Four networks were identified by spatially matching to pre-existing templates (Shirer et al., 2012). The default mode network (DMN) contained five nodes: the ventral anterior cingulate cortex (vACC), dorsal, and ventral posterior conjugate cortex (PCC), and right and left inferior parietal lobules (IPL). The frontoparietal network (FPN) was defined using bilateral superior frontal gyrus (SFG) and angular gyrus (AG). Subcortical (SC) nodes included brain regions having differential group accumulation of [^11^C]PK11195, namely, bilateral putamen and hippocampus. The node time-series were defined as the first principal component resulting from the singular value decomposition of voxels in an 8-mm-radius sphere, which was centered on the peak voxel per each node (Tsvetanov et al., 2016).

After extracting nodal time-series we sought to reduce the effects of noise confounds on functional connectivity effects of node time-series using a general linear model (Geerligs et al., 2017). This model included linear trends, expansions of realignment parameters, as well as average signal in the white-matter and CSF, including their derivative and quadratic regressors from the time-courses of each node (Satterthwaite et al., 2013). The signals in the white-matter and CSF were created by using the average across all voxels with corresponding tissue probability >0.7 in associated tissue probability maps available in the SPM12 software (https://www.fil.ion.ucl.ac.uk/spm/software/spm12/). A bandpass filter (0.0078–0.1 Hz) was implemented by including a discrete cosine transform set in the general linear model, ensuring that nuisance regression and filtering were performed simultaneously (Hallquist et al., 2013; Lindquist et al., 2019). The total head motion for each participant, which was used in subsequent between-subject analysis as a covariate of no interest (Geerligs et al., 2017), was quantified using the approach reported by Jenkinson et al. (2002), i.e., the root mean square of volume-to-volume displacement. Finally, the functional connectivity between each pair of nodes was computed using Pearson's correlation on post-processed time-series.

##### PET protocols and preprocessing.

All participants underwent [^11^C]PK11195 PET imaging to assess the extent and distribution of neuroinflammation while patients with MCI also underwent [^11^C]PiB (Pittsburgh compound-B PET) scanning to evaluate the degree of β-amyloid accumulation. [^11^C]PK11195 and [^11^C]PiB PET were produced with high radiochemical purity (>95%), with [^11^C]PiB PET having a specific activity >150 GBq/μmol at the end of synthesis, whereas [^11^C]PK11195 specific activity was ∼85 GBq/μmol at the end of synthesis. PET scanning used a GE Advance PET scanner (GE Healthcare) and a GE Discovery 690 PET/CT, with attenuation correction provided by a 15 min 68Ge/68Ga transmission scan and a low dose computed tomography scan, respectively. The emission protocols were 550 MBq [11C]PiB injection followed by imaging from 40 to 70 min postinjection, and 75 min of dynamic imaging (55 frames) starting concurrently with a 500 MBq [^11^C]PK11195 injection. Each emission frame was reconstructed using the PROMIS 3-dimensional filtered back projection algorithm into a 128 × 128 matrix 30 cm trans-axial field-of-view, with a trans-axial Hann filter cutoff at the Nyquist frequency (Kinahan and Rogers, 1989). Corrections were applied for randoms, dead time, normalization, scatter, attenuation, and sensitivity.

For [^11^C]PiB we used reference tissue region-of-interest (ROI) defined by ≥90% on the SPM8 gray-matter probability map (smoothed to PET resolution) in the cerebellar cortex (Schuitemaker et al., 2007). For [^11^C]PK11195, supervised cluster analysis was used to determine the reference tissue time-activity curve (Turkheimer et al., 2007). [^11^C]PiB data were quantified using standardized uptake value ratio (SUVR) by dividing the mean CSF corrected radioactivity concentration in each Hammers atlas ROI by the corresponding mean CSF-corrected radioactivity concentration in the reference tissue ROI (whole cerebellum). [^11^C]PiB data were treated as dichotomous measures (i.e., positive or negative) and considered positive if the average SUVR value across the cortical ROIs was >1.5 (Hatashita and Yamasaki, 2010). For [^11^C]PK11195 maps of non-displaceable binding potential (BP_ND_), a measure of specific binding, were determined using a basis function implementation of the simplified reference tissue model, both with and without CSF contamination correction (Gunn et al., 1997). [^11^C]PK11195 BP_ND_ maps (termed from now on *PK maps* for simplicity) were also generated using this basis function approach.

**Figure 1.**
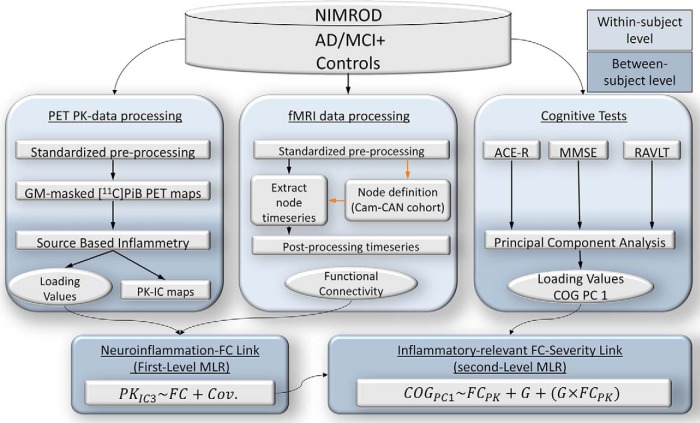
Schematic representation of various modality datasets in the study, their processing pipelines on a within-subject level (light blue), as well as data-reduction techniques and statistical strategy on between-subject level (dark blue) to test for associations between the datasets. FC, Functional connectivity; Cov, covariates; COG PC1, latent variable (cognitive deficit, which summarizes the largest portion of shared variance as the first principal component); GM, gray matter. NIMROD study (Neuroimaging of Inflammation in Memory and Other Disorders), AD/MCI+, Alzheimer's Disease/Mild Cognitive Impairment (positive PET amyloid, 11C PiB PET), PET PK, positron emission tomography [11C]PK11195 ligand (microglia activation), PK-IC maps, PET [11C]PK11195 independent component maps, PK IC3, 3rd component of [11C]PK11195 ligand maps, 11CPiB PET (amyloid PET), fMRI, functional magnetic resonance imaging, Cam-CAN, Cambridge Centre for Ageing and Neuroscience, G, diagnostic group (AD/MCI+ or Controls), ACE-R, Addenbrookes' Cognitive Examination - revised test, MMSE, mini-mental status examination, RAVLT, Ray Audio-Visual Learning Test, MLR, multiple linear regression.

The PK maps were coregistered and warped to the MNI space using the flow fields. To minimize the noise effects from non-gray-matter regions, the normalized PK maps were masked with a group-based gray-matter mask based on voxels having gray-matter tissue probability larger than 0.3 in gray-matter segmented images across all individuals. The normalized images were smoothed using a 6 mm Gaussian kernel. We then used independent component analysis across participants to derive spatial patterns of PK maps across voxels expressed by the group in a small number of independent components. All PK maps were spatially concatenated and submitted to Source Based Inflammetry (SBI) to decompose images across all individuals in a set of spatially independent sources without providing any information about the group (Xu et al., 2009), using the GIFT toolbox. Specifically, the *n*-by-*m* matrix of participants-by-voxels was decomposed into: (1) a source matrix that maps each independent component to voxels (here referred to as PK_IC_ maps), and (2) a mixing matrix that maps PK_ICs_ to participants. The mixing matrix consists of loading values (1 per participant) indicating the degree to which a participant expresses a defined PK_IC_. The independent component loading values for the PK_IC_ were taken forward to between-participant analysis of functional connectivity (Fig. 1), if they were (1) differentially expressed by controls vs. patients with AD pathology; and (2) were associated with atrophy (see Results and Fig. 3). Only one dependent variable (IC3) met these criteria.

#### Statistical analyses

We adopted a two-level procedure, in which, at the first-level, we sought to identify functional connectivity differences associated with differences in [^11^C]PK11195 binding. In a second-level analysis, we tested whether individual variability in functional connectivity (from first-level analysis) is specifically associated with variability in cognitive deficit in the group of patients with AD pathology.

Details about the first-level analysis approach are published previously (Tsvetanov et al., 2018). In short, we used multiple linear regression (MLR) with well conditioned shrinkage regularization (Ledoit and Wolf, 2004) to identify correlated structured sources of variance between functional connectivity and neuroinflammation measures. In particular, this analysis describes the linear relationship between functional connectivity and PK maps on a between-subject level, in terms of structure coefficients (Thompson and Borrello, 1985), by providing a linear combination of the functional connectivity measures, which we term *brain scores*, that are optimized to be highly correlated with the between-subject variability in the expression of the PK maps. Namely, brain-wide connectivity strength for each individual defined the independent variables, and PK_IC_ subject-specific loading values for group differentiating components were used as a dependent variable.

To identify and exclude potential outliers, Grubbs' test was used (Grubbs, 1969; Barnett and Lewis, 1994). None of the loading values in the IC3 was outlying observation. Furthermore, to down-weight the effects of extreme or imprecise data points, the analyses used robust linear regression.

To avoid overfitting, first-level multiple linear regression model was integrated with a fivefold cross-validation approach (Thompson and Borrello, 1985). To minimize the non-negligible variance of traditional *k*-fold cross-validation procedure, we repeated each *k*-fold 1,000 times with random partitioning of the folds to produce an *R* value distribution, for which we report the median value.

Next, we tested the hypothesis that the effect of neuroinflammation on functional connectivity was related to cognitive deficit, in patients relative to controls. To this end, we performed a second-level multiple linear regression (MLR) analysis. Independent variables included subjects' brain scores from first-level MLR (reflecting how strongly each individual expressed the whole-brain pattern of functional connections weighted by the IC3-PET-derived data), group information, and their interaction term (brain scores × group).

In other words, we examined the linear relationship between functional connectivity and expressions of PK maps on a between-subject level, in terms of structure coefficients (Thompson and Borrello, 1985). Hence, we used multiple linear regression to identify a linear combination of the functional connectivity measures, which we term *brain scores*, that were optimized to be highly correlated with the between-subject variability in the expression of PK maps. More specifically, the whole-brain connectivity strength for each individual defined the independent variables (*m* × *n*), and PK_IC_ subject-specific loading values for the group differentiating component (IC3) were used as dependent variable (*m* × *1*), where *m* is the number of subjects and *n* is the number of pairwise connections. This produced a set of structure coefficients (*1* × *n*) and a set of *subject scores* (*m* × *1*) for the functional connectivity data. The structure coefficients reflect the contribution of each connection to the overall functional connectivity pattern; conceptually similar to the component loadings in a PCA on cognitive data and the source matrix in group ICA on PET-PK data (here called SBI). The brain scores indicated how strongly an individual expresses the functional connectivity pattern, which is conceptually similar to the subject-specific scores in a PCA and subject-specific loadings in the SBI analysis. These brain scores were correlated with the subjects' cognitive performance, in a second-level multiple linear-regression analysis. For the cognitive performance, we did not choose a single test, but rather a summarized cognitive ability score in terms of the first principal component across three cognitive tests.

Covariates of no interest included age, sex, head movement, and global gray-matter volume.

## Results

### Source-based inflammetry (SBI)

**Figure 2.**
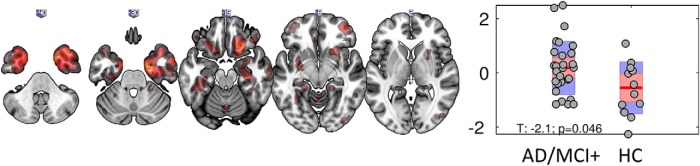
Source Based Inflammetry (SBI) for the component differentially expressed between groups: (left) independent component spatial map reflecting increase in PK ([11C]PK11195 ligand- activated microgia) binding values in cortical and subcortical areas (red blobs) including inferior temporal cortex and hippocampus, these regionally specific increases are over and above the global PK differences between groups. Right, Bar plot of subject loading values for AD/MCI+ and control group (each circle represents an individual) indicating higher loading values for AD/MCI+ than control group as informed by two-sample unpaired permutation test (a robust linear regression was used to down-weight the effects of extreme data points). AD/MCI+, Alzheimer's Disease/Mild Cognitive Impairment (positive PET amyloid, 11C PiB PET), HC, healthy controls.

**Figure 3.**
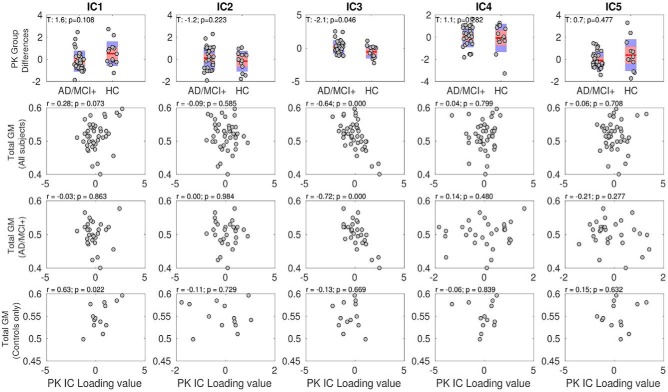
The Source Based Inflammetry (SBI) identified five independent components (ICs) that reflected PK ([11C]PK11195 ligand-activated microgia) binding values in cortical and subcortical areas. The PKIC3 component differed between AD/MCI+ patients and controls (first row, third column). This PKIC3 component negatively correlated with total gray-matter volumes in all individuals as well as in patients only (but not controls only; third column and second, third, and fourth rows). In other words, the patients expressing higher [^11^C]PK11195 binding PKIC3 component (reflecting higher binding in the inferior temporal cortex and hippocampus as shown in Fig. 2) displayed higher levels of brain-wide atrophy. GM, gray matter, AD/MCI+, Alzheimer's Disease/Mild Cognitive Impairment (positive PET amyloid, 11C PiB PET), HC, healthy controlls.

The optimal number of components (*n* = 5) was detected with minimum-distance length criteria. One component showed significant differences between the patient and control group in terms of their loading values (PK_IC3_, *t* = −2.1, *p* = 0.04; Fig. 2 right, robust linear regression). The spatial extent of this PK_IC3_ included voxels with high values in cortical and subcortical regions, including the inferior temporal cortex and hippocampus, indicating that individuals with higher loading values, in this case the patient group, had higher [^11^C]PK11195 binding in these regions, relative to the control group (Fig. 2, left). The other components did not differentiate patients from controls (Fig. 3, first row).

The PK_IC3_ component, which differed between patients and controls, was also the sole PK component that negatively correlated with total gray-matter values in patients but not controls (Fig. 3, third column, and second and third rows). In other words, the patients expressing higher [^11^C]PK11195 binding showed also higher levels of cortical atrophy (Fig. 3, second and third rows). This result was obtained when including the gray-matter volume as a covariate of no interest in the analysis, which suggests that the reported association was over and above the effects of overall brain atrophy.

All in all, our findings imply that the PK_IC3_ component reflects specific patterns of neuroinflammation and neurodegeneration in AD. These patterns were next tested in terms of their relevance for changes in large-scale network function and their interactive effect in mediating cognitive deficit in AD.

### Functional connectivity

**Figure 4.**
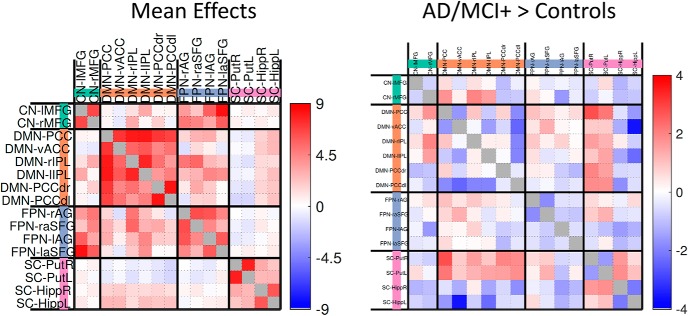
Mean effects (left) and group difference effects (AD/MCI+>Controls, right) between default mode network (DMN) and subcortical (SC) regions using univariate approach. vACC, ventral Anterior cingulate cortex; PCC, posterior cingulate cortex; IPL, intraparietal lobule; FPN, frontoparietal network; Put, putamen; Hipp, hippocampus, AG, angular gyrus; CN-MFG, middle frontal gyrus (cortical network), SFG, superior frontal gyrus; r, right; l, left. Note that the whole pattern of brain connectivity rather than each connection separately was used to study how subject-specific neuroinflammatory levels influence large-scale network connectivity (Fig. 5). AD/MCI+, Alzheimer's Disease/Mild Cognitive Impairment (positive PET amyloid, 11C PiB PET).

As expected, there was strong positive functional connectivity between all nodes within the four networks, identified by spatially matching to pre-existing templates (Fig. 4, left). In terms of group differences, the functional connectivity within networks (within the default mode network, within the frontoparietal network, left-right putamen and left-right hippocampus) and between the default mode network and hippocampus was weaker in patients relative to controls (Fig. 4, right). Furthermore, the connectivity between the putamen and hippocampus increased, whereas the connectivity between default mode network and putamen was less negative for patients relative to controls.

### Functional connectivity and neuroinflammation

**Figure 5.**
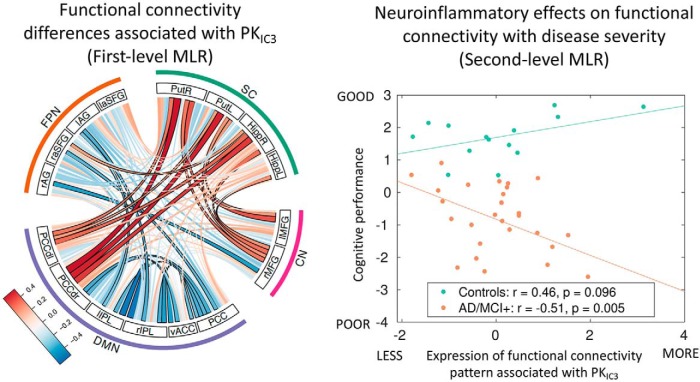
Left, First-level Multiple Linear Regression (MLR) indicating that functional connectivity differences (deviating from groups effects in Fig. 3) are associated positively with PK_IC3_ (PK PET ligand 3rd Independent Component). Connections surviving a threshold of *p* < 0.05 corrected for multiple comparisons are highlighted with a black contour, although it is important to bear in mind that the whole-pattern of brain connectivity was used in the analysis shown on the right. Right, Second-level MLR association between PK_IC3_ pattern of functional connectivity and cognitive performance for patients with AD pathology (including MCI+; orange) and control (green) groups. The group difference in slopes was significant (*p* < 0.0001). By using multiple linear regression and correlations with cognitive performance, we found that the change in patients' cognition was correlated to a pattern of brain connectivity that was itself linked to neuro-inflammation. This relationship between cognition and a PET-rsfMRI association (i.e., neuroinflammation-functional connectivity) was only seen in AD/MCI+ patients, not controls. vACC, ventral Anterior cingulate cortex; PCC, posterior cingulate cortex; IPL intraparietal lobule; FPN, frontoparietal network; SC, subcortical, DMN, default mode network, DMNd, dorsal DMN, Put, putamen; Hipp, hippocampus, AG, angular gyrus; SFG, superior frontal gyrus; R, right; L, left.

The first-level multiple linear-regression model assessing the relationship between PK_IC3_ maps and functional connectivity data was significant (*r* = 0.52, *p* < 0.001). The standard coefficients indicated positive and negative associations between the PK_IC3_ loading values and between-subjects variability in functional connectivity (Fig. 5, left). In other words, individuals with higher [^11^C]PK11195 binding values in the inferior temporal cortex and medial temporal lobe regions (as reflected by higher PK_IC3_ values) showed: (1) increased connectivity between the default mode network, the hippocampus, and other subcortical regions; and (2) weaker connectivity for nodes within the default mode network.

### Linking neuroinflammation, connectivity, and cognitive deficits

The first component of the PCA of cognitive tests explained 80% of the variance across the three cognitive measures (with coefficients of 0.61, 0.61, and 0.52 for MMSE, ACE-R, and RAVLT, respectively).

Next, we tested whether the effects of neuroinflammation on network connectivity were specific to the patient group and whether this functionally-relevant neuroinflammation related to cognitive deficits. Consistently with this hypothesis, the interaction term between group and brain scores (reflecting how strongly each individual expresses the pattern shown in Fig. 5 left, which is the brain-wide pattern of functional connections optimized to highly correlate with the IC3-PET-derived data) was significantly associated to the first component of the PCA of cognitive tests (*t* = −3.4, *p* = 0.004).

A *post hoc* analysis within each group indicated a significant negative association between the behavioral scores from the PCA and functional connectivity/PK-combined indices in the patient group (*r* = −0.51, *p* = 0.005; Fig. 5, right). Conversely, a non-significant positive direction of association for the same relationship between PCA-derived cognitive scores and brain measures was found in controls (*r* = 0.46, *p* = 0.09). The significant difference between patients and controls remains if AD and MCI+ subgroups are analyzed separately (data not shown). The negative association in the patient group indicated that patients in whom higher neuroinflammation was more strongly associated with more abnormal connectivity also performed worse on a summary measure of cognitive deficit.

## Discussion

This study establishes a link between the presence of neuroinflammation and the disruption of large-scale functional connectivity in AD. The degree to which patients expressed the association between abnormal functional connectivity and neuroinflammation itself correlated with their cognitive deficit. In other words, the patients' cognitive scores were correlated to a pattern of brain connectivity that was itself linked to microglia activation. This relationship between cognition and a PET-rs-fMRI association (i.e., neuroinflammation relevant functional connectivity) was only seen in patients with AD, but not healthy controls. This suggests that not only does neuroinflammation relate to large-scale network function, but also that the disruption of connectivity linked to neuroinflammation mediates cognitive deficits in AD.

We propose that the cognitive deficits in AD can be directly related to changes in functional connectivity which in turn are mediated by microglia activation, although we acknowledge that there are several mechanisms by which neuroinflammation can alter brain functional connectivity and vice versa (i.e., the ways in which synaptic firing can influence microglia). Microglia are important contributors in the process of synaptic pruning and regulation of synaptic function (Hong et al., 2016). The microglia's highly mobile and ramified branches can reach and surround synaptic terminals to promote phagocytosis and synaptic demise (Hong et al., 2016). Microglia-induced complement activation might also contribute to synaptic dysfunction and loss, especially in the context of amyloid deposition and neuritic plaque formation (Hong et al., 2016). On the other hand, synaptic firing can influence microglia activation via specific membrane receptors and ion channels (Tofaris and Buckley, 2018).

The anatomical distribution of neuroinflammation in AD and its effects on large-scale network function supports the hypothesis that neuroinflammation might be an early event in the pathogenesis of AD and that our current results are not driven by a global effect of a systemic inflammatory confound which would have affected the whole-brain indistinctively.

Our study has three implications. First, it supports the use of integrative or multimodal neuroimaging as a useful tool to improve our understanding of the brain determinants that mediate healthy and pathological aging (Geerligs and Tsvetanov, 2017).

Second, it reinforces the notion that neuroinflammation is a key pathophysiological mediator of AD and its clinical variability (Weiler et al., 2016). Genome-wide association studies have challenged the idea that neuroinflammation is merely a secondary event caused by neurodegeneration and have conversely sustained a primary role of microglia-related molecular pathways in the etio-pathogenesis of AD (Guerreiro et al., 2013; Jonsson et al., 2013). For instance, mutations in TREM2, an immune cells receptor expressed on microglia, represent a risk factor for AD and other neurodegenerative diseases (Guerreiro et al., 2013; Jonsson et al., 2013). Together with our results, these data suggest that immunotherapeutic strategies might be helpful to reduce the deleterious impact of neuroinflammation on cognitive deficits in AD.

Third, the functional connectivity abnormalities observed here can be considered an intermediate phenotypic expression of the neuroinflammatory pathology in AD. This can be relevant to reconcile the apparent conflict between the encouraging findings from basic research on the role of neuroinflammation in AD pathogenesis (Heppner et al., 2015) and the results from human studies, which as yet have provided little support for immunotherapeutics in AD (Adapt Research Group et al., 2007, 2008), despite epidemiological evidence (Breitner and Zandi, 2001; in't Veld et al., 2001).

In other words, assessing how neuroinflammation influences the intermediate phenotypes of large-scale network functional connectivity might help explaining why clinical trials have failed so far to demonstrate a role for immunotherapeutic strategies because of high patient heterogeneity. We showed marked individual differences in the relationship between resting-state functional connectivity and neuroinflammation in patients with AD at different stages, and it was this variance that was significantly related to individual differences in cognitive performance.

Our study has also limitations and caveats. First, we recognize that even the multivariate methods of statistical associations used here do not in themselves demonstrate causality between neuroinflammation, network dysfunction, and cognition. To address this issue, longitudinal and interventional studies are needed, alongside mediation analyses (Fan et al., 2015; Kreisl et al., 2016).

Second, the molecular pathology of AD is multifaceted, with amyloid deposition, tau accumulation, and vasculopathy. These processes, alone or in combination, may moderate the association between neuroinflammation and functional connectivity; hence, multimodal studies that capture each of these aspects will be useful to formally assess the complex interplay between neuroinflammation, abnormal tau deposition, vasculopathy, and cognitive deficits.

Third, the confounding effect of head motion on functional imaging has been fully recognized as both challenging and critical for interpretation of functional imaging studies, especially in clinical populations. To minimize such confound, we used ME-ICA and validated pre-processing pipelines, which separate changes in the fMRI signal that distinguish between BOLD and non-BOLD signals. Furthermore, we included movement-related parameters as covariates of no interest in second-level analyses, as well as motion and physiological signals in first-level analyses.

Fourth, the use of the [^11^C]PK11195 tracer has its own limitations in terms of reduced affinity to the mitochondrial TSPO in activated microglia, especially compared with second-generation TSPO tracers as PBR28 (Fujita et al., 2017). On the other hand, such second-generation TSPO tracers are affected by common genetic polymorphisms (Owen et al., 2012).

Fifth, at the phenotypic level, it remains to be determined whether the deleterious impact of neuroinflammation on network function can be revealed in pre-symptomatic individuals at risk of AD, for example, in carriers of autosomal dominant genetic mutations. Despite the inclusion of patients with mild cognitive impairment with biomarker evidence of AD pathology, our study cannot resolve the timing of neuroinflammation and its causal relationship to network dysfunction, cell loss, and cognitive deficit. There is initial evidence that neuroinflammation may precede abnormal protein aggregation and brain atrophy in pre-symptomatic carriers of MAPT mutations (Bevan-Jones et al., 2019), although further studies are needed to confirm these preliminary findings in AD or other neurodegenerative diseases.

In conclusion, we have shown that SBI of [^11^C]PK11195 PET data revealed a distributed profile of neuroinflammation in AD, which in turn related to abnormal functional connectivity. Our crossmodal multivariate analyses also indicated that heterogeneity in cognitive status was associated to variability in neuroinflammation-related network dysfunction. These data emphasize the value of multi-modal neuroimaging to study how different aspects of the molecular pathology of AD mediate brain function and cognition. Improved stratification procedures may facilitate more efficient therapeutic trials in AD, based not only on neuro inflammation, tau, atrophy, or connectivity, but on their complex interaction that leads to individual differences in cognitive impairment.
